# Plasma Metabolomic Profiling Suggests Early Indications for Predisposition to Latent Insulin Resistance in Children Conceived by ICSI

**DOI:** 10.1371/journal.pone.0094001

**Published:** 2014-04-11

**Authors:** Alexandra Gkourogianni, Ioanna Kosteria, Aristeidis G. Telonis, Alexandra Margeli, Emilia Mantzou, Maria Konsta, Dimitrios Loutradis, George Mastorakos, Ioannis Papassotiriou, Maria I. Klapa, Christina Kanaka-Gantenbein, George P. Chrousos

**Affiliations:** 1 Division of Endocrinology, Diabetes and Metabolism, First Department of Pediatrics, University of Athens Medical School, Athens, Greece; 2 Metabolic Engineering and Systems Biology Laboratory, Institute of Chemical Engineering Sciences, Foundation for Research and Technology-Hellas (FORTH/ICE-HT), Patras, Greece; 3 Department of Clinical Biochemistry, “Aghia Sophia” Children's Hospital, Athens, Greece; 4 Endocrine Unit, Department of Endocrinology and Metabolism, Evgenidion Hospital, Athens, Greece; 5 Division of In Vitro Fertilization, First Department of Obstetrics and Gynecology, University of Athens Medical School, Athens, Greece; 6 Division of Endocrinology, Second Department of Obstetrics and Gynecology, University of Athens Medical School, Athens, Greece; 7 Graduate Program “Biological Technology”, Department of Biology, University of Patras, Greece; Rutgers University, United States of America

## Abstract

**Background:**

There have been increasing indications about an epigenetically-based elevated predisposition of assisted reproductive technology (ART) offspring to insulin resistance, which can lead to an unfavorable cardio-metabolic profile in adult life. However, the relevant long-term systematic molecular studies are limited, especially for the IntraCytoplasmic Sperm Injection (ICSI) method, introduced in 1992. In this study, we carefully defined a group of 42 prepubertal ICSI and 42 naturally conceived (NC) children. We assessed differences in their metabolic profile based on biochemical measurements, while, for a subgroup, plasma metabolomic analysis was also performed, investigating any relevant insulin resistance indices.

**Methods & Results:**

Auxological and biochemical parameters of 42 6.8±2.1 yrs old ICSI-conceived and 42 age-matched controls were measured. Significant differences between the groups were determined using univariate and multivariate statistics, indicating low urea and low-grade inflammation markers (YKL-40, hsCRP) and high triiodothyronine (T3) in ICSI-children compared to controls. Moreover, plasma metabolomic analysis carried out for a subgroup of 10 ICSI- and 10 NC girls using Gas Chromatography-Mass Spectrometry (GC-MS) indicated clear differences between the two groups, characterized by 36 metabolites linked to obesity, insulin resistance and metabolic syndrome. Notably, the distinction between the two girl subgroups was accentuated when both their biochemical and metabolomic measurements were employed.

**Conclusions:**

The present study contributes a large auxological and biochemical dataset of a well-defined group of pre-pubertal ICSI-conceived subjects to the research of the ART effect to the offspring's health. Moreover, it is the first time that the relevant usefulness of metabolomics was investigated. The acquired results are consistent with early insulin resistance in ICSI-offspring, paving the way for further systematic investigations. These data support that metabolomics may unravel metabolic differences before they become clinically or biochemically evident, underlining its utility in the ART research.

## Introduction

Assisted reproductive technology (ART) comprises the classic *in vitro* fertilization (IVF) and the intra-cytoplasmic sperm injection (ICSI), the latter primarily developed and indicated for the treatment of male infertility [1]–[3]. ICSI bypasses several natural selection barriers, as it involves the injection of a single spermatozoon or even spermatid directly into the oocyte through the *zona pellucida*. The spermatozoa and/or spermatids currently used in ICSI are usually obtained from oligozoospermic men [3]. The ovarian stimulation protocols and the *in vitro* manipulation of both gametes and the blastocyst raise serious concerns about the genetic, epigenetic and developmental risks of the ICSI method to the offspring [4]–[6]. Despite these concerns, the European Society of Human Reproduction and Embryology (ESHRE) reported a recent marked increase in the use of ICSI [1], even in cases of no apparent medical indication [7].

Presently, long-term detailed systematic prospective studies of the ART offspring regarding potential health risks are limited, especially for the ICSI method due to its recent (i.e. in 1992) introduction as ART method. The existing studies have mainly investigated auxological data, indicating a higher risk of preterm delivery and intrauterine growth restriction (IUGR) for the ART offspring, leading to children born prematurely and/or small for gestational age (SGA) [8]. Predisposition for adult disease is *per se* increased by being born SGA [9]–[11], further accentuated by the fact that ART pregnancies are characterized by older mothers, an increased anxiety level before and during pregnancy, and a higher rate of multiple gestations, all risk factors for numerous adverse effects [12]. However, our group as well as others have extended the investigation to cardiometabolic markers reporting that children born after classic IVF show early indices of cardiometabolic derangements, such as higher blood pressure (BP) or higher triglycerides, irrespectively of having been born SGA or not, indicating thus that ART *per se* is a predisposing factor for unfavorable cardiometabolic outcomes [13], [14]. Glucocorticoids are considered the main mediators of the poor long-term outcome of an adverse intrauterine environment, by stimulating hepatic gluconeogenesis, inhibiting insulin actions on skeletal muscle and potentiating insulin's actions on visceral adipose tissue, ultimately promoting visceral adiposity and sarcopenia leading to metabolic syndrome [15]. Recent evidence suggests that ART may be associated with epigenetic modifications in several genes that could have long-term impact on the health of the offspring [16] and that both obesity and metabolic syndrome may have their basis in epigenetic modifications [17]. Thus, systematic studies of the ART offspring are required to validate and investigate the causes of the indicated increased predisposition to unfavorable cardiometabolic profiles later in life. Sensitive holistic methods of the metabolic profile that allow for the early detection of subtle metabolic differences are necessary.

In the systems biology era, high-throughput biomolecular omic analyses of biofluid compositions between two groups of subjects contribute to the identification of characteristically discriminatory multi-component molecular profiles. Compared to singling out specific biomarkers, in multivariate analyses, even subtle differences in a molecular quantity can carry significance if viewed in the context of the observed changes in the rest of the molecules. Thus, there is a higher probability for the identification of considerable variations in molecular profiles in complex medical cases, when conventional analyses fail to provide statistically significant results. Moreover, knowledge-based analysis of high-throughput biomolecular profiles in the context of biological networks lead to a broader perspective of the molecular cell physiology dynamics. In this context, omic analyses pave the way for in-depth systemic studies of human physiology [18]. Metabolomics refers to the analysis of the (relative) concentration profile of the free small metabolite pools of a biological system, providing a comprehensive metabolic signature and a direct link to the phenotype [19]. Untargeted blood metabolomics has been used in the context of disease diagnosis, as biological variation in human blood plasma mainly originates from the constant factors in a person's lifetime, i.e. heritable/familial effects and one's stable individual environment [20], metabolic profile being thus a significant component of the epigenetic fingerprint of an individual. In this context, the usefulness of metabolomics in ART research becomes apparent however there exists currently no published report for application of metabolomics in the analysis of ART offspring physiology.

The objective of this study is to explore in a cross-sectional design the presence of metabolic variation in pre-pubertal children born after ICSI compared to age- and demographically- matched naturally conceived (NC) controls, using medical history and biochemical data, along with untargeted metabolomic analysis of blood plasma in a subgroup of them, investigating any relevance of insulin resistance indices.

## Materials and Methods

### Subjects

#### The “ICSI group”

Forty-two ICSI-conceived [19 boys (45.23%) and 23 girls (54.76%)] healthy prepubertal caucasian children with a mean age of 6.8±2.1 yrs were recruited randomly from the IVF Section of the First Department of Obstetrics-Gynaecology of the University of Athens (Table 1). Underlining the difficulty in recruiting such a large group of ART-conceived children and conducting this type of systematic studies, we report that only 20% of the families contacted were willing to participate in the study.

**Table 1:** Demographic and anthropometric characteristics of ICSI and normal conception (NC) groups.

		ICSI (n = 42)	Control (n = 42)	P
**Gender, n (%)**	**Boys**	19 (45.2%)	19 (45.2%)	0.999^c^
	**Girls**	23 (54.8%)	23 (54.8%)	
**Twins, n (%)**	**Singletons**	13 (31%)	36 (85.7%)	<0.0001^c^
	**Twins**	29 (69%)	6 (14.3%)	
**Pregnancy outcome**	**Uneventful**	22 (52.4%)	27 (71%)	0.09^c^
	**Complicated**	20 (47.6%)	11 (29%)	
**Type of delivery**	**Normal**	5 (11.9%)	17 (40.5%)	0.003^c^
	**Caesarean Section**	37 (88.1%)	25 (59.5%)	
**Perinatal outcome/complications**	**Complicated**	19 (45.2%)	16 (38.1%)	0.5^c^
	**Uneventful**	23 (54.8%)	26 (61.9%)	
**Breastfeeding**	**YES**	30 (73.2%)	30 (71.4%)	0.86^c^
	**NO**	11 (26.8%)	12 (28.6%)	
**Growth for Gestational age**	**SGA**	18 (45%)	13 (34.2%)	0.33^c^
	**AGA**	22 (55%)	25 (65.8%)	
**Gestational age (wk)**		36 (35–37)	38 (37–40)	0.0001^a^
**Birth weight-SDS**		−0.39±0.95	0.42±0.9	0.0001^b^
**Birth Length-SDS**		−0.1 (−0.5–5)	0.5 (−0.3 0.7)	0.015^a^
**Child's Age(yr)**		7.2 (6.1–8.2)	7.2 (5.8–8.2)	0.93^a^
**BMI-sds**		−0.2 (−0.8–0.2)	−0.1 (−0.8–0.5)	0.4^a^
**Height-sds**		0.17±0.92	−0.11±0.99	0.17^b^
**Weight-sds**		−0.09±0.8	−0.03±0.96	0.7^b^
**Waist/height (WHtR)**		0.48 (0.45–0.5)	0.48 (0.44–0.52)	0.5^a^
**Waist/hip (WHR)**		0.96±0.06	0.94±0.06	0.4^b^
**SBP-sds**		0.006±0.9	0.83±1.02	0.0004^b^
**DBP-sds**		−0.5 (−0.7–0.009)	−0.26 (−0.6–0.14)	0.2^a^
**Mother's Age (yr)**		35.6±5.2	31.7±5.3	0.0013^b^
**Father's Age (yr)**		38 (33.5–40.5)	35.5 (31–40)	0.09^a^

Differences between ICSI-Control children using Pearson X^2^ test, two sample Student t-test or Mann-Whitney U test, are depicted and expressed as mean±SD or median and IQR (interquartile range).

a : Mann-Whitney test,

b : Normal distribution, two-sample t-test,

c : Pearson X^2^ test.

Abbreviations: AGA =  Appropriate for Gestational Age; BMI =  body mass index; SBP =  systolic blood pressure DBP =  diastolic blood pressure; ICSI =  Intracytoplasmic Sperm Injection; SGA =  Small for Gestational Age; W/H =  waist-to hip ratio (WHR); WHtR =  Waist-to-height ratio.

#### The “naturally conceived (NC) control group”

Forty-two age- (P = 0.93) and gender- (P = 0.99) matched, naturally conceived healthy prepubertal caucasian children served as controls (Table 1). These children were selected among those referred to the Division of Endocrinology for growth assessment but found to be within the normal growth curves and willing to participate in the study.

From the initial cohort of the 84 children, a subgroup comprising 10 girls from each of the ICSI and NC groups (6.8 ± 2.1yrs) were studied using metabolomics. The girls were selected from the ICSI and NC groups in pairs matched for age, duration of gestation, birth weight and them being singletons or twins (Table 2). We opted for the first investigation of the usefulness of metabolomics in this type of research to be based on a single sex. Girls were selected over boys, since most cases of precocious adrenarche, an insulin resistance marker, occur in females [21], [22]. Thus, we expected for any potential early metabolic variation between the ICSI and NC groups, in the form of differences in the plasma metabolite concentration profiles that were measured through metabolomics, could be more apparent in girls compared to boys, increasing the potential for them being detected even if subtle.

**Table 2:** Demographic and anthropometric characteristics of ICSI and normal conception (NC) girl subgroups used in metabolomics.

		ICSI (n = 10)	Control (n = 9)	P
**Twins, n (%)**	**Singletons**	7 (70%)	6 (66.7%)	0.87^c^
	**Twins**	3(30%)	3 (33.3%)	
**Pregnancy outcome**	**Uneventful**	7 (70%)	6 (75%)	0.8^c^
	**Complicated**	3 (30%)	2 (25%)	
**Type of delivery**	**Normal**	2 (20%)	3 (33.3%)	0.5^c^
	**Caesarean Section**	8 (80%)	6 (66.7%)	
**Perinatal outcome/complications**	**Uneventfull**	7 (70%)	6 (66.7%)	0.87^c^
	**Complicated**	3 (30%)	3 (33.3%)	
**Breastfeeding**	**YES**	3 (30%)	3 (33.3%)	0.87^c^
	**NO**	7 (70%)	6 (66.7%)	
**Growth for Gestational age**	**SGA**	5 (55.6%)	3 (33.3%)	0.34^c^
	**AGA**	4 (44.4%)	6 (66.7%)	
**Gestational age (wk)**		37 (36–39)	38 (36–38)	0.85^a^
**Birth weight-sds**		0.06±1.07	0.12±0.8	0.9^b^
**Birth Length-sds**		−0.14 (−0.26–0.5)	−0.26 (−0.4–0.5)	0.8^a^
**Child's Age (yr)**		6.25 (4.8–7.9)	5.5 (4.3–7.6)	0.6^a^
**BMI-sds**		0.01±0.9	−0.7±0.8	0.08^b^
**Height-sds**		−0.01±0.95	−0.5±0.7	0.2^b^
**Weight-sds**		0.04±0.9	0.095±0.6	0.9^b^
**Waist/height (WHtR)**		0.49±0.04	0.46±0.02	0.12^b^
**Waist/hip (WHR)**		0.96±0.06	0.94±0.04	0.4^b^
**SBP-sds**		−0.5 (−0.7–−0.25)	0.3 (−0.25 to 1.1)	0.1^a^
**DBP-sds**		−0.4±0.6	−0.2±0.3	0.4^b^
**Mother's Age (yr)**		34.1±3.3	33.6±6.1	0.8^b^
**Father's Age (yr)**		37.5 (36–40)	38 (30–39)	0.8^a^

Differences between ICSI-Control children using Pearson X^2^ test, two sample Student *t*-test or Mann-Whitney *U* test, are depicted and expressed as mean±SD or median and IQR (interquartile range).

a :Mann-Whitney *U* test.

b : Normal distribution, two-sample Student *t*-test.

c : Pearson X^2^ test.

Abbreviations: AGA =  Appropriate for Gestational Age; BMI =  body mass index; SBP =  systolic blood pressure DBP =  diastolic blood pressure; ICSI =  Intracytoplasmic Sperm Injection; SGA =  Small for Gestational Age; W/H =  waist-to hip ratio (WHR); WHtR =  Waist-to-height ratio.

### Ethical issues

The study was approved by the Ethics Committee of the “Aghia Sophia” Children's Hospital. Children were included in the study only after informed written consent had been obtained from their parents or guardians.

### Medical History

A thorough medical history was obtained from all subjects and their parents. Specifically, the recorded data included: a) for the parents: maternal and paternal age at the time of conception, years and number of trials of IVF-ICSI in order to achieve a pregnancy, course of gestation, problems during the perinatal period, type of childbirth (vaginal delivery or cesarean section), chronic disease of the parents, b) for the children: gender, gestational age, twins or singletons, birth weight, birth length, head circumference at birth, duration of breastfeeding, as well as the child's personal and family medical history. Children were classified as SGA or AGA when birth weight was <10th or between the 10th and 90th centile, respectively, according to the individual centile calculator at www.gestation.net, which took into account birth weight, gestational age, parity, ethnic group, the child's gender, and the mother's height and weight. The children were examined by the same physicians. Demographic and physical examination data recorded are included in Table 1, including height, weight, head circumference, waist to hip ratio (W/H), BMI, pubertal status and systolic (SBP) and diastolic blood pressure (DBP). Girls with Breast Tanner stage (TS) I and boys with testicular size <4 ml were considered prepubertal, while girls with Breast TS≥II and boys with testicular size ≥4 ml pubertal.

### Blood Sampling

Blood collection of all subjects was carried out at 8 AM after an overnight fast; both plasma and serum samples were collected and stored at −80°C until analysis. Biochemical data were obtained at the “Aghia Sophia” Children's Hospital. Metabolomic data acquisition and analysis of the 20 selected plasma samples was carried out at FORTH/ICE-HT.

### Blood Chemistry

Standard blood biochemical, hematological and hormonal parameters as well as the non-conventional cardiometabolic markers High- sensitivity-Interleukin 6 (hsIL-6), YKL-40 and Hepsidin were assessed by standard methodology using standard equipment. Specifically, blood chemistry including serum glucose, total cholesterol, triglycerides, HDL-Cholesterol, LDL-Cholesterol, uric acid, urea, creatinine, SGOT, SGPT, γGT and iron were determined using the Siemens Advia 1800 Clinical Chemistry System (Siemens Healthcare Diagnostics, Tarrytown, NY, USA). The lipoproteins Apo A-I, Apo-B, Lp(a) and hs-CRP levels were quantified by means of latex-particle-enhanced immunonephelometric assays on the BN ProSpec nephelometer (Dade Behring, Siemens Healthcare Diagnostics, Liederbach, Germany). Serum insulin, cortisol and IGF1 levels were measured using the automated chemiluminescence Siemens ACS180 System Analyzer (Siemens Healthcare Diagnostics, Tarrytown, NY, USA). The intra-assay and interassay coefficients of variation (CV) for all measured variables was <5%, with the exception of the insulin assay, in which the CV was <10%. Complete blood counts were performed using the Siemens-ADVIA 120 whole blood auto-analyzer (Siemens Healthcare Diagnostics, Tarrytown, NY, USA). Ferritin levels were measured with the electrochemiluminescence immunoassay “ECLIA” using the Roche Elescys 2010 immunoassay analyzer (Roche Diagnostics Mannheim, Germany). High- sensitivity-Interleukin (IL)-6 levels were measured by sandwich ELISA (R&D systems, Minneapolis, MN, USA) with a CV <6%. Serum concentrations of YKL-40 were determined by sandwich ELISA (Quidel, Santa Clara, CA, USA) with 3.6% and 5.3% intra- and inter-assay CVs, respectively. Hepcidin levels were measured by sandwich ELISA (DRG Instruments, Marburg, Germany), the intra- and inter-assay CVs, where 4.5% and 6.2%, respectively. Homeostasis model assessment (HOMA), as marker of insulin resistance, was calculated according to the original formula [23].

### Metabolomic Data Acquisition and Normalization

The Gas Chromatography-Mass Spectrometry (GC-MS) metabolomic analysis of 300 µL blood plasma samples was carried out after appropriate adaptation of a previously described protocol [24]. After addition of 0.14 µg ribitol and 0.25 µg [U-^13^C]-glucose as internal standards, each sample was derivatized with 100 µL of 20 mg/mL methoxyamine hydrochloride in pyridine and 200 µL of N-methyl-trimethylsylil-trifluoroacetamide (MSTFA), following the protocol. At least three measurements for each sample were acquired, while the experiment was carried out twice in consecutive weeks. After appropriate data normalization and filtering [24], [25], the profiles of 70 (35 of known identity and 12 of known chemical class) metabolite relative peak areas (RPAs) were considered in the multivariate statistical analysis for the extraction of biologically relevant conclusions. Full description of the data normalization and filtering steps can be found in File S1 and the normalized metabolomic dataset is provided in Table S1.

### Statistical Analyses

Differences of demographic and biochemical data between groups were evaluated by the two sample Student t-test or by the non-parametric Mann–Whitney U-test, according to the normality of the data. The Pearson chi-square (X^2^) test was applied to examine the relations between categorical variables. A *p-value* of <0.05 was considered significant. Analysis was conducted using the STATA-9 statistical software.

Multivariate statistical analysis of the metabolomic and biochemical data of the 20 subject subgroup was carried out using the significance analysis for microarrays (SAM) algorithm in the TM4 MeV (version 4.9.0) omic data analysis software [26], and the Partial Least Squares – Discriminant Analysis (PLS-DA) algorithm in the XLSTAT statistical software (version 2013.4.03). The biochemical profiles included the markers shown in Table 3 augmented by the children's age and BMI. The significant metabolic differences between the groups were visualized by positioning the metabolites, the concentration of which was identified as significantly different between the two groups based on SAM, on the inter-organ metabolic network that was reconstructed based on metabolic databases, human metabolism literature and our own metabolomic data, as described in File S2.

**Table 3:** Biochemical, hormonal and non-conventional cardiometabolic markers in ICSI and NC children groups.

		ICSI (n = 42)	Control (n = 42)	p-value**
**Hematologic parameters**	**Hct (%)**	39.5±2.9	38.4± 2.2	0.07*
	**Hb (g/dL)**	13.1± 1.1	12.8± 0.8	0.22*
	**Fe (µg/dL)**	71.9± 21.7	88.8±38.9	0.1*
	**Ferritin (µg/L)**	35 (20–48)	33 (26–45)	0.7
**Biochemistry**	**Glucose (mg/dL)**	81.5 (77–86)	83 (78–88)	0.36
	**Urea (mg/dL)**	31 (28–34)	34.5 (30–38)	0.04
	**Creatinine (mg/dL)**	0.62± 0.07	0.6±0.08	0.25*
	**SGOT (U/L)**	30 (27–32)	27 (24–32)	0.08
	**SGPT (U/L)**	20 (16–24)	14 (12–19)	<0.00001
	**γ-GT (U/L)**	12 (9–15)	11 (9–13)	0.6
	**Uric Acid (mg/dL)**	3.55±0.75	3.85±0.77	0.07*
	**Total Cholesterol (mg/dL)**	167.7±25.3	172.7±24.5	0.35*
	**TG (mg/dL)**	43 (36–50)	52 (36–66)	0.07
	**HDL (mg/dL)**	63.9±8.9	60.8±12	0.17*
	**LDL (mg/dL)**	94.6± 21.2	100.9±22	0.18*
	**ApoA1 (mg/dL)**	153±21.1	156.1±19.8	0.5*
	**ApoB (mg/dL)**	75.7±14.7	74.2± 14.9	0.65*
	**Lp(a) (mg/dL)**	6.2 (2.4–9.8)	7.2 (3.9–17.3)	0.16
	**T3 (ng/dL)**	182.6±25	156.7±30.1	0.0001*
**Hormones**	**T4 (µg/dL)**	8.7±1.2	8.9±1.3	0.3*
	**TSH (µIU/mL)**	2.99 (2.1–4)	2.4 (1.8–3.7)	0.22
	**Insulin (µIU/mL)**	5.2 (3.9–6.8)	4.75 (4.1–7.8)	0.9
	**Cortisol (µg/dL)**	11.2 (8.5–14.8)	11.5 (9.3–15.8)	0.4
	**IGF-1 (ng/mL)**	159 (112–226)	165 (133–221)	0.58
	**HOMA**	1.04 (0.8–1.4)	0.94 (0.8–1.6)	0.9
**Inflammation markers**	**YKL-40 (ng/mL)**	15.1 (7.3–22.8)	24.58 (16–33.7)	0.0002
	**Hepcidin (ng/mL)**	59.9 (44.2–84.8)	69.2 (52.5–101.7)	0.15
	**hs-IL6 (pg/mL)**	1.2 (0.7–1.9)	1.3 (0.5–1.9)	0.38
	**hsCRP (mg/L)**	0.44±0.04	0.78±0.14	0.022*

Differences are depicted as mean±SD or median and IQR (interquartile range), according to normality of the data.

*normal distribution, Student t-test for two independent samples;

**MannWhitney test.

Abbreviations:ALP =  Alkaline Phosphatase; Apo-A1 =  apolipoprotein-A1; Apo-B =  apolipoprotein-B; hsCRP =  high-sensitivity C-reactive protein; ICSI =  Intracytoplasmic Sperm Injection; IGF1 =  Insulin like Growth Factor 1; LDL =  low-density lipoprotein; Lp(a) =  lipoprotein(a); NC =  naturally conceived; TG =  triglycerides; YKL-40 =  Human cartilage glycoprotein 39; hsIL-6 =  high-sensitivity interleukin-6.

## Results

### Anthropometric & Biochemical Data

In the ICSI group, mothers'age [35.6±5.2 (ICSI) vs. 31.7±5.3 (NC), *p* = 0.0013], prevalence in twins [29 (ICSI) vs. 6 (NC), *p*<0.0001] and cesarean sections [37 (ICSI) vs. 25 (NC), *p* = 0.003] were significantly higher than in NC. Moreover, gestational age [median: 36 wks (ICSI) vs. 38 (NC), *p* = 0.0001], birth weight Standard Deviation Score (SDS) [−0.39±0.95 (ICSI) vs. 0.42±0.9 (NC), *p*<0.0001] and birth length SDS [median: −0.1 (ICSI) vs. 0.5 (NC), *p* = 0.015] were significantly lower in ICSI children than controls. No differences were identified regarding gender, pregnancy outcome, perinatal outcome/complications, breastfeeding, or fathers' age (Table 1).

Statistical analysis of the demographic data indicates a well selected and consistent group with no differences in the family and personal lifestyle or other demographic parameters that may influence the metabolic phenotype between ICSI-children and controls (Table 1). Moreover, from the physical examination, only the SBP-SDS was significantly lower in ICSI than controls (*p* = 0.0004). Similarly, significantly lower values in the non-conventional cardiometabolic markers YKL-40 [median: 15.1 (ICSI) vs. 24.6 (NC), *p* = 0.0002] and hs-CRP [0.44±0.3 (ICSI) vs. 0.78±0.87 (NC), *p* = 0.022] were observed in the ICSI group (Table 3). Among the biochemical and hormonal markers, SGPT and T3 were significantly higher in the ICSI than the NC group. Moreover, urea was lower in the ICSI than the NC group, even though less prominently in the girls' sub-set analyzed using metabolomics (Table 4) compared to the entire group of children (Table 3), due to the high standard deviation of the measurement combined with the smaller number of children in the girls' subset. In the subset of girls analyzed using metabolomics (Tables 2 and 4), only T3 was identified as significantly higher in the ICSI group. Finally, cortisol levels showed a notable decrease in the ICSI compared to the NC group in the subset of girls analyzed using metabolomics, while there was no difference between the two groups over the entire set of children.

**Table 4:** Biochemical, hormonal and non-conventional cardiometabolic markers in ICSI and NC girl subgroups used in metabolomics.

		ICSI (n = 10)	Control (n = 9)	p-value**
**Hematologic parameters**	**Hct (%)**	39 (36.8–39.4)	37.4 (35.4–39.9)	0.9
	**Hb (g/dL)**	13.3 (12.2–13.3)	12.6 (11.8–13.3)	0.5
	**Fe (µg/dL)**	69.8±19.3	83±37.5	0.46*
	**Ferritin (µg/L)**	22.6±12.3	33±9.5	0.15*
**Biochemistry**	**Glucose (mg/dL)**	75.5 (71–82)	79 (78–86)	0.2
	**Urea (mg/dL)**	31.8±6	33.6±7.7	0.6*
	**Creatinine (mg/dL)**	0.57±0.05	0.59±0.06	0.5*
	**SGOT (U/L)**	32.3±7.2	28.1±7.7	0.2*
	**SGPT (U/L)**	18.8±5.6	16.2± 6.5	0.4*
	**γ-GT (U/L)**	8.7±2.3	10.4±1.8	0.09*
	**Uric Acid (mg/dL)**	3.7±1.1	4.1±0.95	0.4*
	**Total Cholesterol (mg/dL)**	170.2±22.7	168.7±22.3	0.88*
	**TG (mg/dL)**	47 (40–57)	45 (38–54)	0.46
	**HDL (mg/dL)**	58±7.6	61.3±12.5	0.48*
	**LDL (mg/dL)**	101.6±18.3	97.3±18.3	0.62*
	**ApoA1 (mg/dL)**	147.3± 20.3	156±26.1	0.4*
	**ApoB (mg/dL)**	78.3± 10.5	75±11.8	0.5*
	**Lp(a) (mg/dL)**	5.8 (3.1–7.6)	17.3 (3.6–33.5)	0.15
	**T3 (ng/dL)**	195.7±27.8	143.5± 25	0.0007*
**Hormones**	**T4 (µg/dL)**	8.9±0.8	9.15±1.2	0.7*
	**TSH (µIU/mL)**	2.86±0.99	2.9±1.4	0.9*
	**Insulin (µIU/mL)**	4.7 (2.4–6.1)	4.3 (4.1–8.2)	0.4
	**Cortisol (µg/dL)**	8.6 (5.9–11.9)	17.9 (11.2–22.3)	0.02
	**IGF-1 (ng/mL)**	130.5 (110–185)	129 (107–165)	0.87
	**HOMA**	0.9 (0.5–1.1)	0.91 (0.7–1.4)	0.5
**Inflammation markers**	**YKL-40 (ng/mL)**	20.4±10.8	21.8±8.2	0.75*
	**Hepcidin (ng/mL)**	68.3 (43.9–89.5)	71.6 (44–119.8)	0.77
	**hs-IL6 (pg/mL)**	1.6 (0.94–1.9)	0.95 (0.6–2.7)	0.44
	**hsCRP (mg/L)**	0.39 (0.18–0.66)	0.48(0.14–1.75)	0.7

Differences are depicted as mean±SD or median and IQR (interquartile range), according to the normality of the data.

*normal distribution, t-test for two independent samples;

**MannWhitney U test.

Abbreviations:ALP =  Alkaline Phosphatase; Apo-A1 =  apolipoprotein-A1; Apo-B =  apolipoprotein-B; hsCRP =  high-sensitivity C-reactive protein; ICSI =  Intracytoplasmic Sperm Injection; IGF1 =  Insulin like Growth Factor 1; LDL =  low-density lipoprotein; Lp(a) =  lipoprotein(a); TG =  triglycerides; YKL-40 =  Human cartilage glycoprotein 39; hsIL-6 =  high-sensitivity interleukin-6.

### Metabolomic analysis

**Figure 1:**
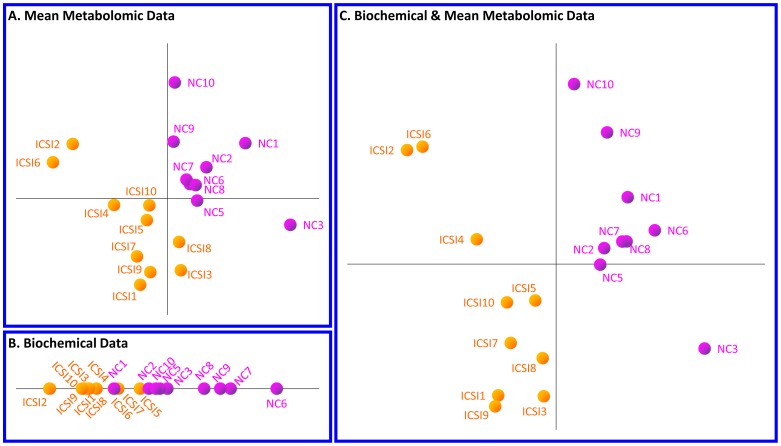
Multivariate statistical analysis of (A) metabolomic, (B) biochemical, (C) combined datasets for the girl subgroup. Partial Least Squares – Discriminant Analysis (PLS-DA) analysis indicates a fair discrimination between the ICSI and NC girl subgroups based on their (A) metabolic profiles, (B) biochemical profiles and (C) combined profiles. In these graphs, each point corresponds, respectively, to the metabolic, biochemical or combined profile of the subject, whose number is shown next to it. The axes of the graphs correspond to functions of multiple variables (i.e. metabolites and/or biochemical markers).

The GC-MS metabolomics data of the 10 ICSI and 10 NC girls were analyzed using PLS-DA to investigate whether the two subgroups can be differentiated based on the 70 metabolite concentration profiles. The PLS-DA method projects the experimental space to a low-dimension perspective corresponding to maximal variance between the two investigated sets of measurements; in the PLS-DA graph, each point corresponds to the metabolic or biochemical profile of a subject. As shown in Figure 1A, the ICSI and NC groups were clearly differentiated based on their GC-MS metabolic profiles. The groups were distinguished based on their biochemical profiles too, even though the discrimination was not as clear as in the metabolomic data analysis (Figure 1B). Notably, the observed difference between the two groups is markedly enhanced when both their metabolic and biochemical profiles were considered (Figure 1C).

**Figure 2:**
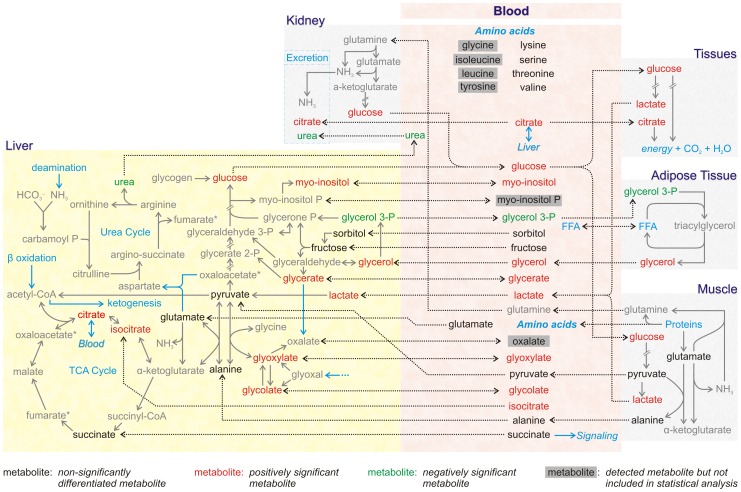
The significant metabolites between the ICSI and NC girl subgroups within the inter-organ metabolic network. Positioning the significant metabolites shown in Table 3 within the reconstructed network indicates metabolic physiology differences between the ICSI and NC girl subgroups. All known metabolites detected in the plasma GC-MS metabolic profiles are shown in the area of the figure named “blood”; among these, the metabolites which were not included in the analysis after the normalization and filtering steps are shown in gray boxes. The names of the positively and negatively significant metabolites (Table 3) are shown in red and green fonts, respectively, while the nonsignificant are shown in black. The plasma metabolites are connected with dashed lines with the same metabolite pool in any of the depicted tissues. The intra-tissue pools of the plasma positively and negatively significant metabolites are shown in red and green, respectively; it is noted that we cannot predict the intra-tissue metabolite concentration from its plasma concentration, but we have tried to include the most significant tissue “sources” and “sinks” that contribute to the observed plasma concentration.

Having established separation of the ICSI and NC groups based on their metabolic profiles, significance analysis (SAM) was applied to identify the metabolites with difference in the concentration characteristic of this separation. SAM is used for the significance analysis of omic data, as it has the advantage of not requiring them to follow a particular distribution. Full description of SAM analysis for the metabolomics and biochemical datasets is provided in File S3. It was found that among the 70 metabolites included in the analysis, 34 (18 of known identity and 9 of known chemical class) and 3 (urea, glycerol 3-phosphate and 1 unknown) metabolites were, respectively, of significantly higher (positively significant) or lower (negatively significant) concentration in the ICSI than the NC group (Table 5). Sugars, sugar alcohols, sugar acids and lipids dominate the list of the positively affected metabolites, with citrate and myo-inositol being the most discriminatory. Moreover, the total concentration of the 70 metabolites was higher in the ICSI group. The difference in the metabolic physiology of the ICSI compared to the NC subjects is visualized in the context of the appropriately color-coded inter-organ metabolic network, in which the names of the known positively and negatively affected metabolites are, respectively, shown in red and green (Figure 2). In accordance with the statistical analysis of biochemical markers discussed above, SAM analysis indicated T3 of significantly larger concentration in the ICSI 10-girl subgroup. The fact that SAM analysis of the metabolomic data indicated urea as the most negatively significant metabolite in the ICSI girls' subgroup compared to NC, while this decrease is not that prominent when only the biochemical measurements are considered, is an example of the significance of the multivariate statistical analysis on a profile of metabolite concentrations compared to the statistical analysis of a single measurement separately. In the biochemical analyses each of the indices is measured independently, with unique experimental handling from the other measurements, and is statistically evaluated in a univariate way. On the other hand, in the metabolomic analysis, the concentrations of the metabolites are measured simultaneously usually with a different protocol compared to the biochemical analysis, and the generated dataset is processed with algorithms of multivariate statistical analysis. Multivariate approaches consider the difference in the concentration of each given metabolite between the two (ICSI and NC) groups in the context of the respective variation of the rest of the metabolites, taking also into consideration the variance of all measurements among the multiple technical replicates considered in the metabolomic analysis. In this way, multivariate analysis can attribute statistical significance to differences in metabolite concentrations for which the standard deviation of the measurements in the two groups could hinder a statistically significant result if evaluated alone, independently of the other data. Therefore, the biochemical and metabolomic approaches differ both in the experimental procedures for metabolite quantification and in the statistical evaluation, which could justify any discrepancies between the two analyses for the common metabolites. As far as the biomedical conclusions are concerned, the two approaches are complementary, as it is shown in our study, as combined the two provide a larger perspective of the physiological state, which cannot be provided from each dataset alone.

**Table 5:** List of positively and negatively significant metabolites in the ICSI compared to the natural conception (NC) group.

Positively significant metabolites	Negatively significant metabolites	Order of Significance
citrate	urea	11^a^
myo-inositol	x_13	22^a^
U_083 (similar to U_088 & U_078)		3
U_041 (sugar acid)		4
U_046 (sugar – hexose)		5
U_010 (putatively α-hydroxyvalerate)		6
glycerate		7
α-tocopherol		8
phosphate		9
U_088 (similar to U_083 & U_078)		10
P2412 (sugar, marker ion 204)		11
(chiro- or) scyllo-inositol		12
U_078 (similar to U_083 & U_088)		13
lactate		14
unknown_no120/P2427 (sugar acid)		15
U_055 (sugar pyranose)		16
glycerol monostearate		17
glucose		18
P2400 (putatively alpha-ketoglutarate)		19
cholesterol		20
gluconate		21
9,12-(z,z)-octadecadienoate (linoleic acid)	*FDR (median) = 0 metabolites*	22
U_039 (sugar pyranose)		23
glyoxylate		24
glycerol		25
threonate		26
U_040 (sugar)		27
U_009 (putatively α-hydroxyisobutyrate)		28
U_014		29
U_048 (sugarpyranose)		30
glycolate		31
U_094 (putatively lipid; same marker ion as stearate)	*FDR (median) = 1.3 metabolites (3.8%)*	32
isocitrate		33
myristate	glycerol 3 –P	343^a^
erythritol	*FDR (median) = 2.2 metabolites (5.85%)*	35

a The first number in the column corresponds to the order of significance of the positively significant metabolites; the second number corresponds to the order of significance of the negatively significant metabolites.

The table shows the list of positively and negatively significant metabolites at three significance threshold cutoffs. Positively/Negatively Significant Metabolites: Metabolites the concentration of which is significantly larger in ICSI compared to the NC group; FDR (median): False Discovery Rate (median) at each significance threshold cutoff, shown in metabolite number and percentage of the total significant metabolite number (in parenthesis).

## Discussion

We report the cardiometabolic profile of a well selected group of ICSI-conceived children with a mean age of 6.8±2.1 yrs in comparison to naturally-conceived age- and gender-matched controls, based on both the “conventional” biochemical and hormonal markers and metabolomic analysis for a subgroup of them. Notably, statistical analysis of the auxological and biochemical dataset for the entire group indicated low urea and low-grade inflammation markers (YKL-40, hsCRP) and high triiodothyronine (T3) in ICSI-children compared to controls. Low urea and high T3 was also validated by the combined analysis of the metabolomic and biochemical profiles of the girl subgroup. The identified significantly higher concentration of T3 in the ICSI group agrees with probable epigenetic changes in the hormonal profile in the ICSI children. Higher concentration of T3 may imply increased peripheral conversion of T4 to T3, already reported in obese subjects [27]. Hyperthyroidism increases the flux of gluconeogenesis, the Cori cycle and adipose tissue lipolysis [28], leading to glucose intolerance and insulin resistance [29]. Interestingly, no elevations of T3 were observed in children conceived by classic IVF; these children rather had elevated TSH with normal T3 and T4 values [30]. Urea was the most significant metabolite with reduced concentration in the ICSI-conceived children, validated very prominently in the metabolomic dataset of the girl subgroup. Urea is synthesized in the liver in order to detoxify the organism from NH4^+^. The reduced urea concentration can be either explained by reduced amino acid breakdown or by nitrogen imbalance with increased disposal of N [31], [32]. Further studies are needed to investigate this observation. The decreased levels of cortisol in the ICSI compared to the NC group within the subset of girls analyzed using metabolomics is consistent with a recent publication reporting lower salivary cortisol levels in the ICSI-conceived girls [33]. Moreover, there is growing evidence of hypothalamo-pituitary-adrenal axis dysfunction and blunted cortisol response in children born preterm or small for gestational age [34]–[35]. Interestingly, studying alone the non-conventional cardiometabolic markers, YKL-40 and hsCRP, would be characterized as improved in the ICSI- compared to the NC group. Specifically, for serum YKL-40, there is a growing body of evidence that its levels are increased in cases of low-grade inflammation. Moreover, it has been recently published that YKL-40 is also increased in obese prepubertal children suggesting that it represents a marker of insulin resistance not only in adulthood but in childhood as well. Therefore, YKL-40 was selected to be measured in our study as a potential marker of inflammation, endothelial dysfunction and diabetes and an independent predictor of overall cardiovascular mortality [36], [37], [38]. However, in the context of the other metabolic differences, the obtained values for YKL-40 and hsCRP could indicate a positive response of a part of the physiology to counteract the negative derangements in other processes, balancing a healthy state for these children. Obviously, negative predisposition to certain cardiometabolic diseases does not mean definite manifestation in adult life, but only in the case that certain environmental stimuli could trigger the particular physiological imbalance.

It should be noted, however, that combining the anthropometric and biochemical approach results with metabolomics analysis in the girl subgroup enabled a more prominent and informative differentiation between the ICSI and NC girls. More than half of the metabolites analyzed were identified with a statistically different concentration between the two groups based on the multivariate dataset significance analysis SAM. Such substantial metabolic difference cannot be viewed as coincidental due to the small number of considered subjects; it has of course to be validated in larger groups of children. The set of metabolites, which were identified with significantly higher concentration in the ICSI compared to the NC group, was dominated by sugars, sugar alcohols, sugar acids and fatty acids, with most of the known having been associated with obesity, insulin resistance and/or metabolic syndrome. The higher concentration of glucose (and other sugars) and lactate in the ICSI group is consistent with a higher activity of the Cori cycle [39], as shown in the reconstructed inter-organ metabolic network, this cycle having been associated with non-insulin-dependent diabetes in obese subjects [40]. The higher concentration of glycerol and four fatty acids (myristate, linoleic acid, glycerol monostearate and unknown U_094) followed by the lower concentration of the glycerol-3-phosphate, imply a higher lipolysis flux in the ICSI than the control group [41]. Higher glycerol and free fatty acid levels in the blood have been implicated in metabolic disorders [42] and obesity [43]. This state is consistent with the observed higher concentration of alpha-tocopherol, the biologically active form of the lipophilic vitamin E [44], which is positively correlated with blood lipid levels [45] and adiposity [46]. Glyoxylate and its precursor glycolate are directly implicated in lipid β-oxidation. These measurements are consistent with the higher concentration of citrate in the ICSI group, the accumulation of which in the post-absorptive state, along with that of isocitrate, represents higher rates of lipid β-oxidation [47]. The presented metabolomic analysis results are the first reported in the context of the ART effect on the cardiometabolic profile of the offspring. It strongly indicates the usefulness of this approach in detecting early indices of metabolic derangements which could contribute to predisposition to metabolic diseases in adult life.

This study has clearly produced indications that ICSI children, like classic IVF-children, have a metabolic profile compatible with insulin resistance. This similarity ends with this though. In contrast to classic IVF children who have increased nonconventional indices of inflammation, ICSI offspring appear to have an improved inflammatory profile [48]. Why and how this will translate in the future is not known. Certainly though it appears that the actual ART procedure employed may have distinct biochemical effects in the offspring.

## Conclusions

The present study contributes a large auxological and biochemical dataset of a consistent group of pre-pubertal ICSI-conceived subjects to the research of the ART effect to the health of the offspring. The importance of this contribution becomes more apparent considering that such systematically collected data are even more sporadic for the ICSI compared to the IVF method. Moreover, it provides the first GC-MS metabolomics dataset for a subgroup of subjects in the context of this type of research. The acquired results support the concerns for increased predisposition of ICSI offspring for cardiometabolic disorders in later life. Moreover, the data demonstrate the value of metabolomic analysis in providing a high resolution perspective of the metabolic state, enabling the determination of characteristic metabolic profiles in complex cases even before impairment can be clearly demonstrated at the biochemical level. Thus, the metabolomics approach could significantly contribute to the research of the ART cardiometabolic effects. The obtained metabolomic findings should be further confirmed in a larger group of children with comparative analysis of both sexes.

## Supporting Information

Table S1 **Normalized Metabolomic Dataset.** This excel file contains the normalized metabolomic dataset of the girl subgroup, which was used in the multivariate statistical analysis.
(XLSX)

File S1 **Metabolomic data normalization and filtering process.** This textfile describes the methods and criteria based on which the normalization and filtering of the metabolomics data was carried out.
(DOCX)

File S2 **Inter-organ metabolic network reconstruction.** This textfile describes which prior biological knowledge reported in the literature and metabolic databases was used to reconstruct an inter-organ metabolic network connecting the metabolites measured in the blood plasma with the metabolic networks of various organs, including liver, muscle, kidney, adipose and other tissues, shown in Figure 2.
(DOCX)

File S3 **Multivariate statistical analysis of metabolomic and biochemical datasets of the girl subgroup.** This file describes the application of the Significance Analysis of Microarrays (SAM) method for the analysis of the metabolomic and biochemical datasets. Figures A and B in File S3 depict, respectively, the SAM graph for the metabolomics and the biochemical datasets.
(DOCX)
