# Supplementary material for: Plasma Metabolomic Profiling Suggests Early Indications for Predisposition to Latent Insulin Resistance in Children Conceived by ICSI
Source: PLoS One. 2014 Apr 11;9(4):e94001. doi: 10.1371/journal.pone.0094001 (PMC3984097; doi:10.1371/journal.pone.0094001)
Supplement: File S1 — Metabolomic data normalization and filtering process. This textfile describes the methods and criteria based on which the normalization and filtering of the metabolomics data was carried out. (DOCX) [file pone.0094001.s002.docx]

**File S1: Metabolomic data normalization and filtering process**

The plasma metabolic profile acquisition for the subgroup of 10 ICSI and 10 NC girls was carried out twice in consecutive weeks [to be called with the extension A (i.e. week 1) and B (i.e. week 2)]. In each week, the metabolic profile of each subject was quantified at least twice [to be called with the extension inj1 (i.e injection 1), inj2 (i.e. injection 2), etc]; for normalization purposes, the profile of NC2 girl was measured eleven (11) times. In this way, the raw dataset included 64 and 57, respectively, metabolic profiles for the 10 ICSI and the 10 NC girls, respectively. The peak identification and quantification was carried out based on the commercial NIST and an in-house peak library. The raw dataset comprised 167 marker ion peak areas corresponding to derivatives of known chemical category (as defined in [[1](#_ENREF_1)] and [[2](#_ENREF_2)]) metabolites.

Metabolomic data validation, normalization and filtering were carried out as described in [[3](#_ENREF_3)]. First, it was verified that all profiles in each experiment were acquired under the same GC-MS operational conditions, based on the constant ratio of the two [U-^13^C] – glucose derivative peak areas; the isotopically labelled glucose was used as internal standard. Second, the relative areas of all identified peaks (RPAs) in each profile were estimated from their normalization with the 319 marker ion peak area of the internal standard ribitol. Plasma samples for which a significant standard deviation (>30%) in the ribitol peak area among the various injections was observed were removed from further analysis; the profiles of NC4 subject fell in this category. In the remaining profiles, a cumulative (effective) RPA was estimated for each known amine group containing metabolite in a profile as the weighted sum of the RPAs of its multiple derivatives. The weights used in this step for each amine group containing metabolite with multiple derivatives (i.e. serine, threonine and valine) were estimated from the metabolic profiles of the C8_A sample, as follows: (a) for serine: w1 = 3.4375, w2 = 6.6036, considering b = 0.43; (b) for threonine: w1 = 2.7917, w2 = 2.9108, considering b = 0.2; (c) for valine, w1 = 0.6455, w2 = 34.4069, considering b = 1.3. Finally, (a) the smallest of the two derivative peaks of the known ketone-group containing metabolites (i.e. glucose-MeOx2, fructose-MeOx1), (b) the peaks corresponding to unknown amine-group containing metabolites, (c) the peaks (i) that were identified as derivatization artefacts, or

(ii) with significant carry over, or (iii) that were inconsistently detected among samples and/or injections of the same sample, or (iv) with significant variation (>27%) between injections, were filtered from the analysis. Moreover, the glucose-MeOx 1 peak and the two glucopyranose RPAs were summed into one, considered as representative of glucose in the plasma samples. Finally, profiles that were identified as significantly increasing the overall variance between the different injections of particular samples were removed from further analysis. To avoid any bias in the data analysis due to the multiple profiles measured for NC2, the 11 profiles were grouped into five profiles as follows: injections 1-2, injections 3-4, injections 5-6, injections 7-8 and injections 9-11 were, respectively, grouped into profiles named finally as NC2_A_inj1, NC2_A_inj2, NC2_A_inj3, NC2_inj4, NC2_A_inj5.

After this stage of normalization and data filtering, 44 and 42, respectively, profiles of 70 metabolites for 9 NC and 10 ICSI girls were considered for further analysis, after the profiles of the subjects in the two weeks were normalized with respect to the mean value of each respective metabolite in both weeks. Specifically, the RPA of a metabolite m in a profile j of week i (i = A or B),
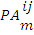
 , was multiplied by
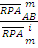
 , where the numerator represents the mean RPA of the metabolite in both weeks and the denominator represents the mean RPA value of the metabolite in the respective week *i* (A or B). For the samples for which only profiles from one week remained after the filtering step, this normalization ratio was equal to 1. Finally, any missing metabolite RPA values in any of the profiles were replaced by the mean RPA value for this metabolite in the other injections of the same sample. In the rare occasion that RPA values were missing for a metabolite in all injections of a sample, then these were replaced by the mean RPA value of the metabolite in the other samples of the same group (NC or ICSI). In this way, the mean RPA value for a metabolite in a particular group (NC or ICSI) remained unchanged after these replacements.

The final normalized metabolic profile dataset that was used in data analysis is shown in Table S1.

**REFERENCES**

1. Kanani H, Chrysanthopoulos PK, Klapa MI (2008) Standardizing GC-MS metabolomics. J Chromatogr B Analyt Technol Biomed Life Sci 871: 191-201.

2. Kanani HH, Klapa MI (2007) Data correction strategy for metabolomics analysis using gas chromatography-mass spectrometry. Metab Eng 9: 39-51.

3. Spagou K, Theodoridis G, Wilson I, Raikos N, Greaves P, et al. (2011) A GC-MS metabolic profiling study of plasma samples from mice on low- and high-fat diets. J Chromatogr B Analyt Technol Biomed Life Sci 879: 1467-1475.
