# Supplementary material for: Plasma Metabolomic Profiling Suggests Early Indications for Predisposition to Latent Insulin Resistance in Children Conceived by ICSI
Source: PLoS One. 2014 Apr 11;9(4):e94001. doi: 10.1371/journal.pone.0094001 (PMC3984097; doi:10.1371/journal.pone.0094001)
Supplement: File S2 — Inter-organ metabolic network reconstruction. This textfile describes which prior biological knowledge reported in the literature and metabolic databases was used to reconstruct an inter-organ metabolic network connecting the metabolites measured in the blood plasma with the metabolic networks of various organs, including liver, muscle, kidney, adipose and other tissues, shown in Figure 2. (DOCX) [file pone.0094001.s003.docx]

**File S2: Inter-organ metabolic network reconstruction**

In this study, we had to analyze the plasma metabolic profile of prepubertal girls at the post-absorptive state. The latter is characterized by gut emptiness and utilization of energy from body stores, i.e. glycogen and fat. To relate the plasma metabolic profile to the metabolic physiology of the children to be able to extract biologically relevant conclusions, we had to reconstruct the inter-organ metabolic network based on available information in the literature, metabolic databases and our metabolomic data. Below, we combine information about the metabolic physiology of the post-absorptive state that we collected from the literature and assisted us in the metabolic network reconstruction.

At the post-absorptive state, tissues take up glucose from the blood to metabolize it. The consumed circulating glucose should be replenished by the liver through glycogenolysis or gluconeogenesis; it is considered that each route contributes half of the total glucose production in the liver at the post-absorptive state [[1](#_ENREF_1),[2](#_ENREF_2)]. The recycling of glucose and lactate is described as the Cori cycle [[3](#_ENREF_3)].

Glucose produced from glycerol originates from the breakdown of fatty acids (FA) in adipose tissue. If FA were to form triacylglycerol for storage, glycerol should be converted to glycerol 3- phosphate in the liver and be transferred to adipose tissue for FA biosynthesis, as the activity of glycerol kinase is highest in the liver [[4](#_ENREF_4)]. In the post-absorptive state, instead, lypolysis is more active than lypogenesis and there is a net release of FA and glycerol from adipose tissue [[5](#_ENREF_5),[6](#_ENREF_6)]. Glycerol 3-phospahte can be converted to glycerone-phosphate [4] and contribute to gluconeogenesis [[7](#_ENREF_7)]. Glycerol can also be oxidized to glyceraldehyde, which in turn is phosphorylated to glyceraldehyde 3-P [[8](#_ENREF_8)], another intermediate product of gluconeogenesis. Glycerone-phosphate and glyceraldehyde may also be produced from the breakdown of fructose 1-phosphate, which originates from phosphorylation of fructose [[8](#_ENREF_8),[9](#_ENREF_9)]. Except from its uptake from blood, fructose also originates from the oxidation of sorbitol through the action of sorbitol dehydrogenase, an enzyme found present in all rat tissues with highest mRNA levels in the liver [[10](#_ENREF_10)] and also detected in human liver [[8](#_ENREF_8)]. Aldehyde dehydrogenase and glycerate kinase are responsible for the conversion of glyceraldehyde to glycerate and then to glycerate 2-phosphate, respectively, but the activity of glycerate kinase is significantly low in the human liver, this having been proposed as a reason for the accumulation of glycerate in the case of its increased production [[8](#_ENREF_8)]. Glycerate metabolism has also been coupled to glyoxalate/oxalate and hydroxypyruvate metabolism (not shown in the reconstructed metabolic network) [[11](#_ENREF_11),[12](#_ENREF_12)].

In the post-absorptive liver, the amino acids may also serve as substrates for gluconeogenesis. The main origin of amino acids in the blood is the protein breakdown occurring inside muscle cells [[13](#_ENREF_13)]. However, the relative concentration of each amino acid does not reflect the composition of muscle proteins [[14](#_ENREF_14)]. In fact, glutamine and alanine are the most abundant amino acids in blood samples [[15](#_ENREF_15)]. Amino acids also serve as signaling and regulatory molecules [[16](#_ENREF_16)]. Regarding their use in the energy metabolism alanine and glutamine are the two amino acids mainly used in the energy metabolism at the post-absorptive state [[17](#_ENREF_17)], each being involved in different metabolic and physiological processes. Alanine in muscles originates largely from the transamination of pyruvate [[18](#_ENREF_18),[19](#_ENREF_19)], while it accounts for half of the total hepatic amino acid uptake [[20](#_ENREF_20)]. The liver does contain active alanine transaminase [[21](#_ENREF_21)], being able to regenerate pyruvate to be used for gluconeogenesis [[22](#_ENREF_22)].

Gluconeogenesis is active in the kidney too; actually, the glucose production in kidney and liver are equal per gram of tissue [[23](#_ENREF_23)]. Glutamine may be a substrate for gluconeogenesis in the kidney, but is also coupled with nitrogen metabolism. Glutaminase and glutamate dehydrogenase deaminate glutamine and glutamate, respectively (glutamate may also be transaminated, not shown), generating alpha-ketoglutarate, used for glucose production, and ammonia, involved in acid-base homeostasis [[17](#_ENREF_17),[24](#_ENREF_24),[25](#_ENREF_25)]. However, in the liver, deamination of glutamine and glutamate is directly associated with urea biosynthesis [[20](#_ENREF_20)], as any ammonia released in hepatocytes is readily fixed into urea [[26](#_ENREF_26),[27](#_ENREF_27)]. The intestine is also a fair site of ammonia production [[26](#_ENREF_26),[28](#_ENREF_28)]. Periportal hepatocytes are responsible for urea synthesis, while any nitrogen in the form of ammonia that escapes from these cells is fixed to glutamine in the perivenous hepatic cells before entering the systemic circulation (not shown)[[27-29](#_ENREF_27)].

In blood plasma, citrate is the most abundant TCA cycle intermediate and can cross cell membranes. High levels of intracellular citrate can slow down glycolysis and this metabolite can be used as substrate for FA biosynthesis indicating high activity of energy metabolisms [[30](#_ENREF_30)]. However, at the post-absorptive state there is a net breakdown of FA at the organism level [[5](#_ENREF_5)] and the high concentration of citrate should be associated with increased levels of lipolysis. In the kidney, the use of citrate is associated with acid-base homeostasis [[31](#_ENREF_31)]. In addition, divalent ion physiology is in cohesion with citrate metabolism, as it can chelate Fe2+, Ca2+ and Zn2+ ions [[32](#_ENREF_32)].

Regarding the metabolism of glyoxylate and oxalate synthesis, glyoxal is a major precursor of glycolate and glyoxylate in humans and originates from a variety of physiological sources, e.g. autoxidation of carbohydrates and ascorbate, degradation of glycated proteins and lipid peroxidation [[33](#_ENREF_33)]. Serine and glycine are also significant entry points of carbon into this pathway [[34](#_ENREF_34)] and oxalate synthesis is directly linked with glyoxylate metabolism [[35](#_ENREF_35)] predominantly in the liver [[36](#_ENREF_36)].

**REFERENCES**

1. Landau BR, Wahren J, Chandramouli V, Schumann WC, Ekberg K, et al. (1996) Contributions of gluconeogenesis to glucose production in the fasted state. J Clin Invest 98: 378-385.

2. Chandramouli V, Ekberg K, Schumann WC, Kalhan SC, Wahren J, et al. (1997) Quantifying gluconeogenesis during fasting. Am J Physiol 273: E1209-1215.

3. Cori CF (1981) The glucose-lactic acid cycle and gluconeogenesis. Curr Top Cell Regul 18: 377-387.

4. Lin EC (1977) Glycerol utilization and its regulation in mammals. Annu Rev Biochem 46: 765-795.

5. Stich V, Berlan M (2004) Physiological regulation of NEFA availability: lipolysis pathway. Proc Nutr Soc 63: 369-374.

6. Frayn KN, Humphreys SM (2012) Metabolic characteristics of human subcutaneous abdominal adipose tissue after overnight fast. Am J Physiol Endocrinol Metab 302: E468-475.

7. Patsouris D, Mandard S, Voshol PJ, Escher P, Tan NS, et al. (2004) PPARalpha governs glycerol metabolism. J Clin Invest 114: 94-103.

8. Heinz F, Lamprecht W, Kirsch J (1968) Enzymes of fructose metabolism in human liver. J Clin Invest 47: 1826-1832.

9. Knight J, Assimos DG, Easter L, Holmes RP (2010) Metabolism of fructose to oxalate and glycolate. Horm Metab Res 42: 868-873.

10. Estonius M, Danielsson O, Karlsson C, Persson H, Jornvall H, et al. (1993) Distribution of alcohol and sorbitol dehydrogenases. Assessment of mRNA species in mammalian tissues. Eur J Biochem 215: 497-503.

11. Williams HE, Smith LH, Jr. (1971) Hyperoxaluria in L-glyceric aciduria: possible pathogenic mechanism. Science 171: 390-391.

12. Booth MP, Conners R, Rumsby G, Brady RL (2006) Structural basis of substrate specificity in human glyoxylate reductase/hydroxypyruvate reductase. J Mol Biol 360: 178-189.

13. Cynober LA (2002) Plasma amino acid levels with a note on membrane transport: characteristics, regulation, and metabolic significance. Nutrition 18: 761-766.

14. Ruderman NB (1975) Muscle amino acid metabolism and gluconeogenesis. Annu Rev Med 26: 245-258.

15. Le Boucher J, Charret C, Coudray-Lucas C, Giboudeau J, Cynober L (1997) Amino acid determination in biological fluids by automated ion-exchange chromatography: performance of Hitachi L-8500A. Clin Chem 43: 1421-1428.

16. Meijer AJ, Dubbelhuis PF (2004) Amino acid signalling and the integration of metabolism. Biochem Biophys Res Commun 313: 397-403.

17. Stumvoll M, Perriello G, Meyer C, Gerich J (1999) Role of glutamine in human carbohydrate metabolism in kidney and other tissues. Kidney Int 55: 778-792.

18. Chang TW, Goldberg AL (1978) The metabolic fates of amino acids and the formation of glutamine in skeletal muscle. J Biol Chem 253: 3685-3693.

19. Chang TW, Goldberg AL (1978) The origin of alanine produced in skeletal muscle. J Biol Chem 253: 3677-3684.

20. Meijer AJ, Lamers WH, Chamuleau RA (1990) Nitrogen metabolism and ornithine cycle function. Physiol Rev 70: 701-748.

21. Yang RZ, Park S, Reagan WJ, Goldstein R, Zhong S, et al. (2009) Alanine aminotransferase isoenzymes: molecular cloning and quantitative analysis of tissue expression in rats and serum elevation in liver toxicity. Hepatology 49: 598-607.

22. Consoli A, Nurjhan N, Reilly JJ, Jr., Bier DM, Gerich JE (1990) Contribution of liver and skeletal muscle to alanine and lactate metabolism in humans. Am J Physiol 259: E677-684.

23. Mitrakou A (2011) Kidney: its impact on glucose homeostasis and hormonal regulation. Diabetes Res Clin Pract 93 Suppl 1: S66-72.

24. Tapiero H, Mathe G, Couvreur P, Tew KD (2002) II. Glutamine and glutamate. Biomed Pharmacother 56: 446-457.

25. Curthoys NP (2001) Role of mitochondrial glutaminase in rat renal glutamine metabolism. J Nutr 131: 2491S-2495S; discussion 2496S-2497S.

26. Watford M (1993) Hepatic glutaminase expression: relationship to kidney-type glutaminase and to the urea cycle. FASEB J 7: 1468-1474.

27. Curthoys NP, Watford M (1995) Regulation of glutaminase activity and glutamine metabolism. Annu Rev Nutr 15: 133-159.

28. Spanaki C, Plaitakis A (2012) The role of glutamate dehydrogenase in mammalian ammonia metabolism. Neurotox Res 21: 117-127.

29. Brosnan JT (2003) Interorgan amino acid transport and its regulation. J Nutr 133: 2068S-2072S.

30. Mycielska ME, Patel A, Rizaner N, Mazurek MP, Keun H, et al. (2009) Citrate transport and metabolism in mammalian cells: prostate epithelial cells and prostate cancer. Bioessays 31: 10-20.

31. Simpson DP (1983) Citrate excretion: a window on renal metabolism. Am J Physiol 244: F223-234.

32. Chypre M, Zaidi N, Smans K (2012) ATP-citrate lyase: a mini-review. Biochem Biophys Res Commun 422: 1-4.

33. Lange JN, Wood KD, Knight J, Assimos DG, Holmes RP (2012) Glyoxal formation and its role in endogenous oxalate synthesis. Adv Urol 2012: 819202.

34. Rodda BE, Chernish SM, Nash JF (1976) A pharmacokinetic method for designing prolonged-release formulations--propoxyphene hydrochloride. J Pharmacokinet Biopharm 4: 243-253.

35. Robijn S, Hoppe B, Vervaet BA, D'Haese PC, Verhulst A (2011) Hyperoxaluria: a gut-kidney axis? Kidney Int 80: 1146-1158.

36. Farinelli MP, Richardson KE (1983) Oxalate synthesis from [14C1]glycollate and [14C1]glyoxylate in the hepatectomized rat. Biochim Biophys Acta 757: 8-14.
