# Supplementary material for: Plasma Metabolomic Profiling Suggests Early Indications for Predisposition to Latent Insulin Resistance in Children Conceived by ICSI
Source: PLoS One. 2014 Apr 11;9(4):e94001. doi: 10.1371/journal.pone.0094001 (PMC3984097; doi:10.1371/journal.pone.0094001)
Supplement: File S3 — Multivariate statistical analysis of metabolomic and biochemical datasets of the girl subgroup. This file describes the application of the Significance Analysis of Microarrays (SAM) method for the analysis of the metabolomic and biochemical datasets. Figures A and B in File S3 depict, respectively, the SAM graph for the metabolomics and the biochemical datasets. (DOCX) [file pone.0094001.s004.docx]

**File S3: Multivariate statistical analysis of metabolomic and biochemical datasets of the girl subgroup**

The Significance Analysis for Microarrays (SAM) is tailored for high-throughput (omic) biomolecular datasets. The advantage of this analysis over Student t-test or other similar significance analysis algorithms is that SAM does not require the data to follow a specific distribution. This assumption could be significantly erroneous for the omic data, which at each molecular level of cellular function are connected through the corresponding biomolecular network (e.g. metabolic, signalling etc) structure. In a SAM graph of this study each point corresponds to a particular metabolite or biochemical marker (see Figures A and B in File S3, respectively). The axes of the graph correspond to the measured value (y axis) and expected value (x axis) of the metabolite (or biochemical marker) concentrations; the expected value refers to the case in which the difference between the two compared groups with respect to the metabolic (or biochemical) profiles would have been based only on random error. The same color dashed lines forming a belt around some points of the graph define a threshold of significance δ_i_; FDR (median) depicts the false discovery rate (%) for the particular δ_i_ over the metabolites (or biochemical markers) considered in this analysis. If the difference between the measured and the expected value for a metabolite (or biochemical marker) concentration is in absolute value larger than δ_i_, then the metabolite (or biochemical marker) concentration is considered as significantly different between the two studied groups (i.e. ICSI and NC groups in our study). Now if this difference is (a) positive, the metabolite (or biochemical marker) is, in our study, of significantly higher concentration in the ICSI compared to the NC group (called throughout the text as positively significant and shown in red in Figures A and B of File S3) or (b) negative, then the metabolite (or biochemical maerker) is, in our study, of significantly lower concentration in the ICSI compared to the NC group (called throughout the text as negatively significant and shown in green in Figures A and B in File S3).The further a significant point is from the origin (0,0) of the SAM graph (and thus the further from the dashed lines of the significant threshold), the higher the statistical significance of the metabolite (or the biochemical marker).

Performing SAM on the metabolomics dataset (see respective SAM curve in Figure A in File S3), it indicated 37 out of the 70 metabolites with significantly different concentration (to be called “significant metabolites) in the ICSI compared to the NC girl group at the most “lenient” significance threshold δ_5_=1.00 with a number of false positives - False Discovery Rate (FDR)-median – equal to 6.56% x 70 = 2.43 metabolites. At the strictest significance threshold δ_1_= 1.45 at which the FDR (median) is equal to zero, the number of significant metabolites drops to 24. The full list of significant metabolites at the two extreme significance thresholds with indication for an intermediate threshold are shown in Table-3 in reducing order of significance.

SAM was also performed on the biochemical dataset for the same ICSI and NC girl subgroup considered in the metabolomic analysis (see respective SAM curve in Figure B of File S3). This dataset comprised the measurements of the biochemical markers shown in Table 2 augmented by the children’s age and BMI. The only biochemical marker clearly identified as significantly different between the ICSI and NC groups was hormone T3, having statistically larger concentration in the former than in the latter group.


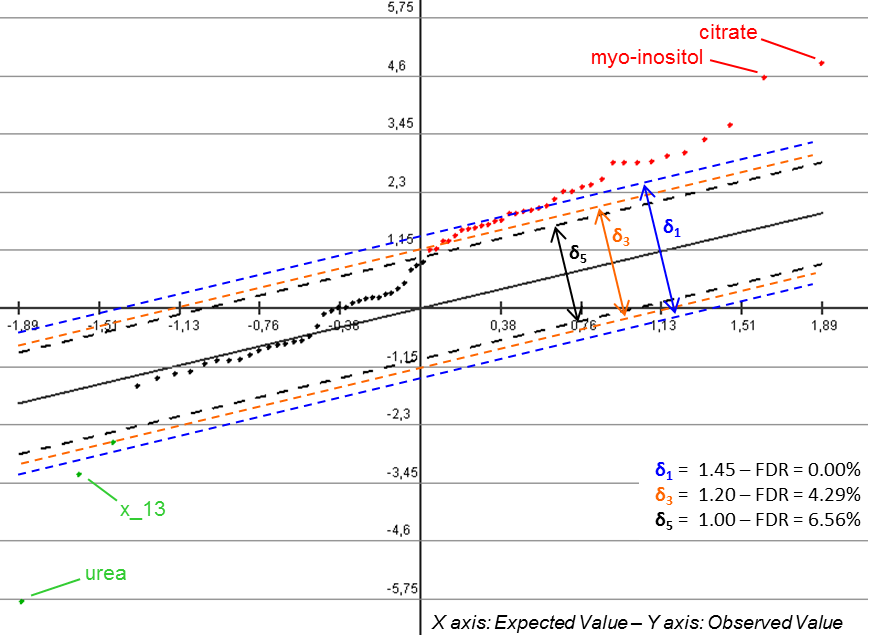


ICSI vs. NC

**Figure A in File S3: SAM curve of the metabolic profile data of the ICSI compared to the NC girl subgroups**. In this graph, we show three different thresholds of significance, starting from the strictest (for FDR equal to zero, blue lines) ending at the most lenient (black lines), indicating an intermediate threshold too (orange lines). In all cases, the FDR remains significantly low, which shows that there is a clear difference between the two groups with respect to their metabolic profiles. The list of the significant metabolites for each significance threshold depicted in Figure A in File S4 is shown in Table 3 in decreasing order of significance. Based on the rule discussed above, citrate and myo-inositol are the most discriminant positively significant metabolites, with urea and unknown x_13 being the most discriminant negatively significant metabolites. The rest of the positively and negatively significant metabolites shown in Table 3 correspond to the red and green, respectively, points in this graph that follow the most discriminant as we move from the outsides to the origin of the graph.


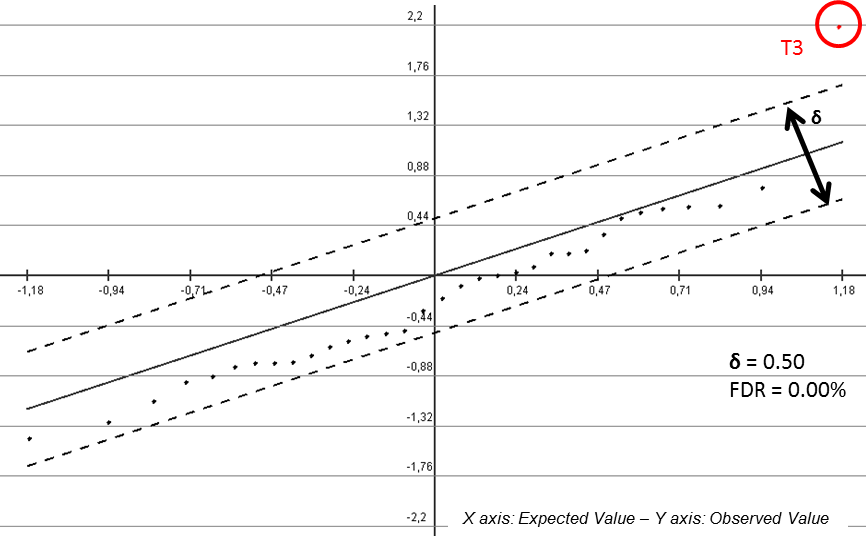


ICSI vs. NC

**Figure B in File S4: SAM curve of the biochemical profile data of the ICSI compared to the NC girl subgroups considered in the metabolomic analysis**.
